# A Protocol to Evaluate Retinal Vascular Response Using Optical Coherence Tomography Angiography

**DOI:** 10.3389/fnins.2019.00566

**Published:** 2019-06-12

**Authors:** David Cordeiro Sousa, Inês Leal, Susana Moreira, Sónia do Vale, Ana S. Silva-Herdade, Patrício Aguiar, Patrícia Dionísio, Luís Abegão Pinto, Miguel A. R. B. Castanho, Carlos Marques-Neves

**Affiliations:** ^1^Ophthalmology Department, Hospital de Santa Maria, Centro Hospitalar Universitário Lisboa Norte, Lisbon, Portugal; ^2^Vision Sciences Study Center, CECV, Faculdade de Medicina, Universidade de Lisboa, Lisbon, Portugal; ^3^Manchester Royal Eye Hospital, Manchester University Hospitals NHS Foundation Trust, Manchester, United Kingdom; ^4^Respiratory Medicine Department, Hospital de Santa Maria, Centro Hospitalar Universitário Lisboa Norte, Lisbon, Portugal; ^5^Endocrinology Department, Hospital de Santa Maria, Centro Hospitalar Universitário Lisboa Norte, Lisbon, Portugal; ^6^Endocrinology Department, Faculdade de Medicina, Universidade de Lisboa, Lisbon, Portugal; ^7^Instituto de Bioquímica, Instituto de Medicina Molecular, Faculdade de Medicina, Universidade de Lisboa, Lisbon, Portugal; ^8^Medicine I Department, Centro Hospitalar Universitário Lisboa Norte, Lisbon, Portugal; ^9^Clinica Universitária de Medicina I, Faculdade de Medicina, Universidade de Lisboa, Lisbon, Portugal

**Keywords:** optical coherence tomography angiography, retinal vascular response, retinal superficial plexus, retinal deep plexus, hypoxia challenge test, handgrip test, autonomic nervous system

## Abstract

**Introduction:**

Optical coherence tomography angiography (OCT-A) is a novel diagnostic tool with increasing applications in ophthalmology clinics that provides non-invasive high-resolution imaging of the retinal microvasculature. Our aim is to report in detail an experimental protocol for analyzing both vasodilatory and vasoconstriction retinal vascular responses with the available OCT-A technology.

**Methods:**

A commercial OCT-A device was used (AngioVue^®^, Optovue, CA, United States), and all examinations were performed by an experienced technician using the standard protocol for macular examination. Two standardized tests were applied: (i) the hypoxia challenge test (HCT) and (ii) the handgrip test, in order to induce a vasodilatory and vasoconstriction response, respectively. OCT-A was performed at baseline conditions and during the stress test. Macular parafoveal vessel density of the superficial and deep plexuses was assessed from the *en face* angiograms. Statistical analysis was performed using STATA v14.1 and *p* < 0.05 was considered for statistical significance.

**Results:**

Twenty-four eyes of 24 healthy subjects (10 male) were studied. Mean age was 31.8 ± 8.2 years (range, 18–57 years). Mean parafoveal vessel density in the superficial plexus increased from 54.7 ± 2.6 in baseline conditions to 56.0 ± 2.0 in hypoxia (*p* < 0.01). Mean parafoveal vessel density in the deep plexuses also increased, from 60.4 ± 2.2 at baseline to 61.5 ± 2.1 during hypoxia (*p* < 0.01). The OCT-A during the handgrip test revealed a decrease in vessel density in both superficial (55.5 ± 2.6 to 53.7 ± 2.9, *p* < 0.001) and deep (60.2 ± 1.8 to 56.7 ± 2.8, *p* < 0.001) parafoveal plexuses.

**Discussion:**

In this work, we detail a simple, non-invasive, safe, and non-costly protocol to assess a central nervous system vascular response (i.e., the retinal circulation) using OCT-A technology. A vasodilatory response and a vasoconstriction response were observed in two physiologic conditions—mild hypoxia and isometric exercise, respectively. This protocol constitutes a new way of studying retinal vascular changes that may be applied in health and disease of multiple medical fields.

## Introduction

In the central nervous system, the possibility of direct visualization of the vascular system is unique to the retina vessels, which derive from the ophthalmic artery, the first branch of the internal carotid artery. (Riva et al., 2016). As one of the most metabolically active tissues in the body, the retina requires an effective blood flow regulation for its normal functioning (Wei et al., 2018). It has the ability for local autoregulation, which is important to keep blood flow relatively constant despite the variations in perfusion pressure (Arjamaa and Nikinmaa, 2006).

The impairment of the normal retinal vascular response is reported in the early stages of a number of ocular diseases, such as diabetic retinopathy (Nguyen et al., 2009; Pemp et al., 2009; Yau et al., 2012; Ramm et al., 2016), age-related macular degeneration, and glaucoma (Garhöfer et al., 2004; Gugleta et al., 2013). Therefore, the study of retinal vessel behavior and blood flow regulatory function is crucial to increase our knowledge about the mechanisms behind several ocular vascular diseases.

A number of non-invasive methods have been used to for retinal vessels’ assessment, including laser Doppler velocimetry (Riva et al., 1985), laser Doppler flowmetry (Riva, 2001; Riva et al., 2010), laser speckle flowgraphy (Tamaki et al., 1994), blue-field entoptoscopy (Riva and Petrig, 1980; Fallon et al., 1985), and color Doppler imaging (Stalmans et al., 2011; Abegao Pinto et al., 2012). However, these devices and techniques are not widely available in clinic, being mostly limited to research purposes (Wang and Luo, 2017; Wei et al., 2018).

Optical coherence tomography angiography (OCT-A) is a novel diagnostic tool with increasing applications in ophthalmology clinics. OCT-A technology uses infrared wavelengths to provide non-invasive, high-contrast, high-resolution imaging of the retinal microvasculature (Koustenis et al., 2017; Spaide et al., 2017; Wei et al., 2018). This technology is an extension of the widely used optical coherence tomography (OCT) and generates images of unprecedented detail by interferometrically measuring the amplitude and delay of reflected or backscattered light from moving erythrocytes. It does so by detecting motion contrast produced by moving blood cells in retinal vessels. Retinal blood flow induces a change between sequential B-scans, while no-flow areas produce no variation. Since no motion in the retina other than blood flow is expected, stationary objects will not produce a significant change in sequential images, while moving objects produce a detectable change. By comparing changes over time, the generated final image clearly defines retinal microvasculature. Recent advances in projection artifact removal allowed researchers to accurately define the deep retinal vascular layers and not only the superficial plexus, overcoming one of its main limitations (Garrity et al., 2017). Its potential for clinical use is tremendous; not only does it allow clinical evaluation of vascular pathologies without the need for invasive procedures, but it can also allow quantitative assessment of the retinal vascular bed. Furthermore, it unlocks new possibilities in detecting functional changes subjects with no visible structural defects.

Our research group has previously reported the potential of OCT-A to detect changes in retinal vessels, having recently published a proof of concept in healthy volunteers to characterize the physiologic retinal vascular response under hypoxic conditions. This work confirmed the ability of this technology to non-invasively detect a significant retinal vasodilatory response to a mild hypoxic stress, in a healthy cohort (Sousa et al., 2017).

Given the reported ability of OCT-A to assess dynamic retinal vascular changes, this manuscript aims to report in detail a protocol for analyzing both vasodilatory and vasoconstriction retinal vascular responses to a standard stimulus with the widely used clinically available OCT-A technology.

## Materials and Methods

### Ethics and Informed Consent

This research protocol follows the tenets of the Declaration of Helsinki (Carlson et al., 2004) and was submitted and approved by the Ethics Committee of Lisbon Academic Medical Center in March 2018. Written informed consent was obtained from all the participants before enrolment, after detailed explanation of the objectives, procedures, and risks of the study. Two standardized tests were applied: i) the hypoxia challenge test (HCT; Vohra and Klocke, 1993) and ii) the handgrip test (Ewing et al., 1985).

As recommended by the Ethics Committee, in order to minimize ethical concerns regarding the HCT, patients and volunteers recruited must had the intention to fly in the future. All the safety recommendations regarding the handgrip test were also followed and the test was stopped if necessary (Ewing et al., 1985). Only the physicians had access to each subject’s electronic health records. Medical confidentiality was assured. By agreeing to be part of this study, all the participants had access to a comprehensive ophthalmological exam. At any time, enrolled subjects could anonymously withdraw from the study.

### Participants

Twenty-four healthy volunteers were recruited. An anonymous questionnaire was carried out, including the following questions: age, gender, smoking-pack years, known diseases and current chronic medication, previous intraocular surgery or trauma, symptoms during previous flights, and intention to fly in the future. Subjects were also asked to abstain from alcohol and caffeine for at least 6 h before the study to reduce the possible autonomic effects and measurement bias (Vinader-Caerols et al., 2012) and were instructed to rest for 10 min in a sitting position before the protocol start.

Exclusion ophthalmological criteria were as follows: the presence of significant lens opacities (Lens Opacities Classification System III equal to or higher than stage 2), high refractive error (spherical equivalent below −6.50 or above +4.00 diopters), history of glaucoma or ocular hypertension, neuro-ophthalmic disease, and previous intraocular surgery. Exclusion systemic criteria included the following: hypertension (defined as systolic blood pressure higher than 140 mmHg and diastolic blood pressure higher than 90 mmHg or use of anti-hypertensive drugs), nephropathy or other documented microvascular complication, diabetes mellitus, local or systemic inflammatory diseases, those taking vasoactive drugs, and smokers of more than 20 cigarettes a day. Pregnant women were excluded.

### Protocol

Firstly, the study protocol was explained individually to every subject, a written consent was given, and the questionnaire was filled. Then, a complete ophthalmological examination was conducted to all subjects, including best-corrected visual acuity, slit-lamp biomicroscopy with fundoscopy, auto-refractometer (RK-5^®^, Canon Europe^®^, The Netherlands), fundus photography (CR-2^®^, Canon, United States), intraocular pressure, and eye biometry (Lenstar^®^, Haag-Streit, Switzerland). Other baseline measurements performed included arterial pressure (Carescape^®^V100, GE Healthcare, Portugal) and pulse oximetry. Room temperature was kept at 22°C, and similar mesopic conditions were adopted throughout the study.

#### Optical Coherence Tomography Angiography

A commercial OCT-A device was used (AngioVue^®^, Optovue, CA, United States), which has an A-scan rate of 70,000 A-Scan/s with 5-μm axial resolution and uses the split-spectrum amplitude-decorrelation angiography (SSADA) algorithm, thus enhancing signal-to-noise ratio of flow detection. The device used also included the latest projection artifact removal algorithm, allowing for a more precise deep plexus analysis.

All examinations were performed by an experienced technician at the required timepoints using the standard protocol for macular examination. Two repeated scans were performed at baseline and during the stress test, respectively. Vessel density of the superficial and deep plexuses was assessed from the *en face* angiograms, by analyzing a predefined annulus with an outer diameter of 3 mm and an inner diameter of 1 mm, corresponding to the parafoveal region. This variable was automatically gathered using AngioAnalytics^®^, the built-in software of the OCT-A device, as a ratio of the white pixels to the total number of pixels (i.e., the proportion of the image occupied by retinal vessels; Yu et al., 2015). Only high-quality images (high signal strength, focused, and without movement artifacts) were considered.

#### Vasodilatory Response—Hypoxia Challenge Test

The vasodilatory response with retinal blood flow increase in response to a decreased arterial oxygen value has already been reported as a physiologic response, mainly associated with the local release of hypoxia-related metabolites, such as retinal relaxing factor, prostacyclin, and lactate (Pournaras et al., 2008; Cheng et al., 2016).

The following protocol was designed in order to comparatively characterize with OCT-A the retinal vessel density changes induced under hypoxia conditions using HCT as a hypoxic stress test. The HCT is performed at sea level in order to create a normobaric hypoxic environment by reducing FiO_2_ and making it equivalent to the flight cabin values. The British Thoracic Society (BTS) proposes a practical and inexpensive protocol to perform HCT (Vohra and Klocke, 1993). Briefly, participants had to breathe FiO_2_ of 15% by using a gas mixture with a supply of 99.993% nitrogen (Linde Healthcare^®^, Portugal) through a 40% flow Venturi mask (Intersurgical^®^, United Kingdom) at 10 L/min. Cardiorespiratory monitoring during HCT was performed using a polygraph and an oximeter in a hand finger (Alice PDX, Philips-Respironics^®^, United States). The parameters monitored during HCT were oxygen peripheral saturation, arterial pressure, and continuous electrocardiography.

As established by the BTS, the recommended HCT duration to obtain stable conditions is 20 min. Accordingly, OCT-A was performed at baseline and then, again, 30 min after HCT start, under the described hypoxic conditions. Then, the Venturi mask and cardiorespiratory monitoring devices were withdrawn. All symptoms were recorded, and the test was stopped if medically necessary.

#### Vasoconstrictive Response—Handgrip Test

The handgrip test, as an isometric exercise, is a sympatheticomimetic test causing steady and safe increases in heart rate and arterial pressure. The associated physiologic retinal vascular response consists in a vasoconstriction response (Ewing et al., 1985; Blum et al., 1999; Zhang et al., 2012).

The following protocol was conducted after having been previously explained in detail to all participants. Subjects sat in a chair in front of the OCT-A device, with the forearm in neutral position, the elbow flexed at 90°, and the wrist with the thumb facing upward. The participants were asked to hold a Jamar hydraulic dynamometer, and maximal grip force (MGF) was calculated with the dominant arm. Systemic blood pressure was monitored in the contralateral arm. The participants were then instructed to relax and place the chin in the OCT-A chinstrap and be prepared for examination. When ready, a voice signal requested the participant to keep a steady contraction of at least one-third of the maximal calculated force (monitored by an investigator). After 90 s, the OCT-A acquisition started, completed for both eyes within the 3- to 5-min handgrip test. The arterial pressure in the contralateral arm was measured every minute and registered. According to the handgrip test recommendations (Ewing et al., 1985), if a diastolic blood pressure higher than 120 mmHg and/or any adverse symptom was registered, the test was interrupted. The procedure was repeated after a 15-min resting time if any reliability-limiting situation occurred.

### Statistics

Statistical analysis was performed using STATA v14.1. A repeated-measures ANOVA model was used to assess differences between the baseline and stress measurements. The Shapiro–Wilk and skewness/kurtosis test suggested the normal distribution of the variables considered, and the inexistence of significant outlier values was also confirmed. Equality of variances was investigated, and the results were reported accordingly, applying the Greenhouse–Geisser correction when variables’ variances were not equal. *p* < 0.05 was considered for statistical significance. To guarantee independence of observations, only the right eye of each patient was considered for analysis.

## Results

### Demographics and Baseline Data

Twenty-four eyes of 24 healthy subjects (10 male) were studied. Mean age was 31.8 ± 8.2 years (range, 18–57 years). Mean best-corrected visual acuity was 0.0 LogMar, mean intraocular pressure was 13.3 ± 2.1 mmHg (range, 10–18 mmHg), with a mean spherical equivalent of −1.3 ± 1.8 D (range, −5 to 2 D) and mean axial length of 24.12 ± 0.9 mm (range, 22.5–25.9 mm). Mean body mass index was 22.6 ± 3.0 kg/m^2^ (range, 18.6–28.7 kg/m^2^).

### Vasodilatory Response—Hypoxia Challenge Test

The peripheral oxygen saturation decreased from 98 ± 1% to stable minimum values of 87 ± 2% during HCT (Table 1). The mean parafoveal vessel density in the superficial plexus increased from 54.7 ± 2.6 in baseline conditions to 56.0 ± 2.0 in hypoxia (*F*_1,23_ = 15.69, *p* < 0.001). The mean parafoveal vessel density in the deep plexuses also increased, from 60.4 ± 2.2 at baseline to 61.5 ± 2.1 during hypoxia (*F*_1,23_ = 16.26, *p* < 0.001) (Table 1). The increase in vessel density was observed in 22 (92%) of the 24 eyes in both plexuses.

**Table 1:** Systemic variables and retinal vascular response to the hypoxia challenge test.

		Baseline	Hypoxia	*p*-value
SAP (mmHg)		118 ± 11	114 ± 10	0.15
DAP (mmHg)		75 ± 9	75 ± 10	0.29
MAP (mmHg)		89 ± 9	88 ± 9	0.97
Heart rate (bpm)		64 ± 8	76 ± 12	0.03
O_2_ Hb saturation (%)		98 ± 1	87 ± 2	<0.0001
Parafoveal vessel density	Superficial plexus	54.7 ± 2.6	56.0 ± 2.0	<0.001
	Deep plexus	60.4 ± 2.2	61.5 ± 2.1	<0.001

*Mean values and standard deviations are presented. bpm, beats per minute; DAP, diastolic arterial pressure; Hb, hemoglobin; MAP, mean arterial pressure; SAP, systolic arterial pressure.*

### Vasoconstrictive Response—Handgrip Test

As depicted in Table 2, the handgrip test was associated with an expected heart rate and systolic and diastolic blood pressure increase compared to baseline (all *p* < 0.001).

**Table 2:** Systemic variables and retinal vascular response to the handgrip test.

		Baseline	Handgrip	*p*-value
SAP (mmHg)		117 ± 12	150 ± 18	<0.0001
DAP (mmHg)		78 ± 10	102 ± 14	<0.0001
MAP (mmHg)		91 ± 10	118 ± 15	<0.0001
Heart rate (bpm)		64 ± 8	78 ± 10	<0.0001
Parafoveal vessel density	Superficial plexus	55.5 ± 2.6	53.7 ± 2.9	<0.0001
	Deep plexus	60.2 1.8	56.7 2.8	< 0.0001

*Mean values and standard deviations are presented. Bpm, beats per minute; DAP, diastolic arterial pressure; MAP, mean arterial pressure; SAP, systolic arterial pressure.*

The OCT-A during the handgrip test revealed that isometric exercise elicited a decrease in vessel density in both superficial (55.5 ± 2.6 to 53.7 ± 2.9, *F*_1,23_ = 27.37, *p* < 0.0001) and deep (60.2 ± 1.8 to 56.7 ± 2.8, *F*_1,23_ = 27.90, *p* < 0.0001) parafoveal plexuses (Table 2). The decrease in vessel density was observed in 22 (92%) and 23 (96%) of the 24 eyes in the superficial and deep plexuses, respectively.

Regarding the systemic response, the mean percent increase in mean arterial pressure and heart rate was 32 ± 1.7% and 23 ± 1.7%, respectively. Table 2 summarizes both OCT-A and cardiovascular response findings to the handgrip test.

## Discussion

As an energy-demanding tissue, the retina blood flow autoregulatory mechanisms are crucial to keep blood flow relatively constant despite the variations in perfusion pressure (Arjamaa and Nikinmaa, 2006; Wei et al., 2018). In this study with OCT-A, we report a standardized, reliable, and non-invasive way of studying retinal vascular vasodilatory and vasoconstrictive responses to hypoxia and isometric exercise, respectively.

Despite the constant blood flow thought to be provided by the choroidal circulation, retinal vessels are believed to present a large reserve for vasodilation and vasoconstriction in order to balance changes in the arterial pressure of oxygen (Geiser et al., 2000; Cheng et al., 2016). The encountered vasodilatory response under mild hypoxic conditions is consistent with the findings of previous studies using different technologies, such as blue-field entoptic phenomenon and scanning laser Doppler flowmetry (Fallon et al., 1985; Strenn et al., 1997). The stress test used in our study, HCT, consistently induced the expected systemic responses, with an increase in heart rate accompanying the hypoxemia. A few OCT-A studies have been published reporting the vasoconstrictive response to hyperoxia (Xu et al., 2016; Hagag et al., 2018). However, to the best of our knowledge, our group was the first to report the vasodilatory response to hypoxia, using the standardized HCT as the hypoxic stimulus.

Although it is believed that autonomic innervation of retinal vasculature is not significant (Pournaras et al., 2008; McDougal and Gamlin, 2015), the vasoconstrictive response to isometric exercise was clearly observed in our study. This response was associated with the expected sympathetic nervous system induced increase in arterial blood pressure and heart rate. As previously described, this regulation should be, at least partially, induced by the local response to the increase in arterial pressure—the Bayliss effect in retinal autoregulation (Robinson et al., 1986; Blum et al., 1999). This observed vasoconstrictive response in retinal vessels is similar to the one described in peripheral arteries’ behavior (Ewing et al., 1985). However, with still so much to unveil about mechanisms behind the retinal autoregulation, we are not able to definitely attribute this retinal vascular response to a specific factor.

For both retinal vascular responses, the differences observed in our sample were striking and quite homogeneous, with more than 90% of the subjects presenting a similar vessel density change in both plexuses. This reinforces the potential of the above-described protocol as a repeatable method for the evaluation of retinal vascular function, in a healthy retina and possibly in a pathologic vascular setting as well. Although the clinical significance of the statistically significant findings encountered should be discussed, the magnitude of change we encountered is comparable to other OCT-A studies. For example (Simonett et al., 2017) were able to distinguish a healthy cohort from patients with no or mild diabetic retinopathy with changes in vessel density similar in magnitude to the ones reported in our study.

In a recent study, Hagag et al. have demonstrated a reduction on the flow index and vessel density (−7.8%) of the deep capillary plexus only and in the flow index of the all-plexus slab measured by OCT-A with hyperoxia. These authors describe an approach to induce hyperoxia that consists in fitting a simple face mask and giving supplemental oxygen for 10 min at a flow rate of 15 L/min, which delivers 60–90% oxygen in the inspired oxygen, therefore creating a systemic hyperoxic condition (Hagag et al., 2018). In contrast, in our work, in order to induce vasoconstriction, we used the handgrip test. We found a significant decrease in the vessel density of both superficial and deep plexus, although with a lower magnitude of change, as detailed in Table 2. The small sample in the work of Hagag et al. and the fact that another vasoconstrictor stimulus (with a different physiologic mechanism) was chosen may have contributed to this difference. These results should be validated by performing both tests (induced hyperoxia and handgrip tests) and comparing the retinal vascular response with OCT-A technology.

Regarding the vasodilatory response, an alternative method that could be considered to induce retinal vessel dilation is the hypercapnia test, as described elsewhere (Raurich et al., 2008, 2009). However, compared to our choice to induce a safe level of hypoxia, this test involves re-inhalation of expired air by inserting a length of corrugated tube between the Y-piece and the endotracheal tube, which increases the deadspace by a volume similar to the tidal volume (V_T_) obtained with a pressure support of 7 cmH_2_O (Raurich et al., 2009). Comparatively, we believe that our work presents a simpler, safer, and reproducible methodology to induce a detectable vasodilation in the retina using OCT-A.

After establishing a protocol to evaluate retinal vascular response with OCT-A, it will be possible to study not only the healthy eye but also responses in a compromised vascular setting, such as in diabetic retinopathy, age-related macular degeneration, and glaucoma. We hypothesize that local factors may play a crucial role when it comes to the retinal vascular regulation and responses in metabolic diseases. In fact, a recent thesis (Eliasdottir, 2018) has suggested that retinal autoregulation is mostly due to myogenic and metabolic factors in order to accommodate local blood flow to differences in perfusion pressure and metabolic needs. In this context, we think that this protocol, once established, could be used to assess functional changes in the retinal vascular physiology before clinically detectable diseases, taking the paradigmatic example of DR.

In summary, this work details a simple, non-invasive, safe, and non-costly method to assess vascular changes in healthy subjects that can be the stepping stone for several experiments. It has gone some way toward enhancing our understanding of possible and reproducible methods to induce and detect vasoconstriction and vasodilation in retinal vessels. Because OCT-A technology and devices are increasingly used in ophthalmology, we hope that our research will serve as an encouragement for the development of technology capable of dynamically assessing the vascular response in opposition to static images only. In fact, and contrary to other devices with mainly research purposes, OCT-A technology is easy to use and increasingly available in ophthalmology clinical practice worldwide (Sadda, 2017) and therefore we think that establishing a protocol with an available tool will have an impact in the neurosciences community.

### Limitations

As a first limitation of our study, we should mention the relatively small sample size, which limits association analysis with demographic and ophthalmic features of the participants. Secondly, the current OCT-A technology is still evolving and one should be careful in the interpretation of the findings and their magnitude. As an example, with the current device, we are not able to estimate absolute blood flow values, but only *perfused vessel densities*. Thirdly, in both protocols, there is an autonomic nervous system activation and, therefore, we are not able to isolate the factor inducing the retinal vascular response from the autonomic-related vascular consequences. Moreover, despite reporting statistical significance in the parafoveal vessel density means in response to both the hypoxic and handgrip tests, we are unsure of the clinical significance of these differences. In fact, there are no current data in literature to indicate what would normal inter-exam variation be when calculating parafoveal vessel density. To be able to comment more accurately about the obtained results, it is essential to know the error associated with the built-in software and then be able to distinguish clinical change from measurement variability. Assessing the reproducibility of these measurements would then be a next step to overcome this caveat (Bland and Altman, 1996). With this study, we expect to promote the development of the technology in order to allow for larger studies to confirm our results with a dynamic (and continuous, ideally) analysis of retinal vascular responses in health and disease, with potentially relevant diagnostic and therapeutic implications.

## Conclusion

This study on human volunteers constitutes a proof of concept on how to evaluate a central nervous system response (i.e., the retinal circulation) in two physiologic conditions—hypoxia and isometric exercise. The importance of identifying such a response with a rapid, non-invasive, and reliable technology used in clinical practice—OCT-A—may be a stepping stone for new lines of research not only in ophthalmology, but also in physiology and neuroscience.

## Data Availability

The datasets generated for this study are available on request to the corresponding author.

## Ethics Statement

This research protocol follows the tenets of the Declaration of Helsinki (Carlson et al., 2004) and was submitted and approved by the Ethics Committee of Lisbon Academic Medical Center in March 2018. Written informed consent was obtained from all the participants before enrolment, after detailed explanation of the objectives, procedures and risks of the study. Two standardized tests were applied: (i) the hypoxia challenge test (HCT; Vohra and Klocke, 1993), and (ii) the handgrip test (Ewing et al., 1985).

## Author Contributions

All authors were involved in the design of the study, contributed to the manuscript writing, and approved the submitted version. DS, IL, SM, SdV, PD, LP, and CM-N were specifically involved in participants’ recruitment, coordination of the study logistics, and preparation and conduction of the protocol. SM and PD were responsible for the hypoxia challenge test protocol. PA provided the equipment and gave support and know-how regarding the handgrip test. DS and CM-N applied the test.

## Conflict of Interest Statement

The authors declare that the research was conducted in the absence of any commercial or financial relationships that could be construed as a potential conflict of interest.
